# Host movement dominates the predicted effects of climate change on parasite transmission between wild and domestic mountain ungulates

**DOI:** 10.1098/rsos.230469

**Published:** 2024-01-03

**Authors:** Eleanor R. Dickinson, Christopher McFarland, Carole Toïgo, D. Michael Scantlebury, Philip A. Stephens, Nikki J. Marks, Eric R. Morgan

**Affiliations:** ^1^ School of Biological Sciences, Queen's University Belfast, 19 Chlorine Gardens, Belfast BT9 5DL, UK; ^2^ Office Français de la Biodiversité, 5 allée de Bethléem, ZI Mayencin 38610, Gières, France; ^3^ Conservation Ecology Group, Department of Biosciences, Durham University, South Road, Durham DH1 3LE, UK

**Keywords:** Alpine ibex, multi-host system, domestic sheep, GLOWORM model framework, elevation, gastrointestinal nematodes

## Abstract

Climate change is shifting the transmission of parasites, which is determined by host density, ambient temperature and moisture. These shifts can lead to increased pressure from parasites, in wild and domestic animals, and can impact the effectiveness of parasite control strategies. Understanding the interactive effects of climate on host movement and parasite life histories will enable targeted parasite management, to ensure livestock productivity and avoid additional stress on wildlife populations. To assess complex outcomes under climate change, we applied a gastrointestinal nematode transmission model to a montane wildlife–livestock system, based on host movement and changes in abiotic factors due to elevation, comparing projected climate change scenarios with the historic climate. The wildlife host, Alpine ibex (*Capra ibex ibex*), undergoes seasonal elevational migration, and livestock are grazed during the summer for eight weeks. Total parasite infection pressure was more sensitive to host movement than to the direct effect of climatic conditions on parasite availability. Extended livestock grazing is predicted to increase parasite exposure for wildlife. These results demonstrate that movement of different host species should be considered when predicting the effects of climate change on parasite transmission, and can inform decisions to support wildlife and livestock health.

## 1. Introduction

Parasites are a major determinant of host survival, fecundity and behaviour, impacting individuals and populations [1–4]. Many parasite species infect both livestock and wildlife in areas where they overlap [5], which can threaten the fitness and viability of wildlife populations and have significant economic implications for livestock keepers [1]. To manage parasite cross-transmission effectively in these systems, we must understand the ecological and environmental drivers of infection and have frameworks readily available to investigate system-specific interactions. The invisible nature of internal parasites and their subclinical impacts on fitness, and technical and logistic challenges to data collection at a scale and resolution sufficient to be informative, are major barriers to empirical understanding of parasite dynamics in natural and shared wild–domestic systems [6]. Consequently, transmission of parasites at the livestock–wildlife interface and their impacts on wildlife populations are prone to be ignored by managers, who have limited tools with which to evaluate and address the issue.

The successful transmission of parasites is driven by both ecological and environmental determinants; including host density and movement [7,8], and changes in environmental conditions, which are the dominant drivers of the availability of infective parasite stages [9]. Interactions between host and environment can alter transmission dynamics, for example through host range shifts or altered migration patterns under climate warming [10–13]. Together these factors may impact the diversity and dynamics of wildlife diseases and parasites through altered zones of climatic suitability and shifts in host presence and contact, across a range of scales [14,15].

Predictive models offer a way to identify the risks of parasite transmission and to design effective interventions, especially in areas that may be difficult to access, and thus data deficient [16,17]. Such areas occur globally, often linked to limited economic resources, making modelling solutions more valuable [18–20]. Most models are parameterised on livestock and for single host systems, but there is scope to extend them to multi-host systems, including wildlife [5,18,19,21]. Transmission of parasites between Alpine ibex, *Capra ibex ibex* and livestock (e.g. domestic sheep, *Ovis aries*) living at high elevations in the central European Alps has not been investigated due to challenges in conducting studies in this environment; however, it is likely to occur, due to the practice of transhumance farming in the Alps and shared parasite communities, particularly gastrointestinal nematodes (GINs) [22,23]. *Teladorsagia circumcincta* has been recorded as one of the most common GIN parasites infecting ibex [22,24,25], and is among the most common and significant parasites in livestock, especially sheep in temperate zones [23,26]. It is necessary to understand the dynamics of parasite spillover between wildlife and livestock, in both directions, and interactions with host movement and the environment, when planning management strategies [27].

Using a combined empirical and mechanistic approach, we modelled parasite transmission in a mountain study system with seasonal vertical movements of ibex and sheep, using historic and projected future climate data. The main aims were to: (i) determine the GIN transmission cycle in ibex, which graze areas at different altitudes through the year; (ii) evaluate the extent and timing of transmission of GINs between ibex and livestock at these different altitudes; (iii) predict the effect of changes in climatic conditions, acting through both host movement and parasite biology, on the transmission cycle of GINs between ibex and livestock; (iv) synthesize findings to recommend how interventions in livestock might serve to attenuate impacts of parasites on livestock and ibex populations; and (v) create an open model framework that can be adapted to other systems.

## 2. Methods

### 2.1. Study area and population

**Figure 1.**
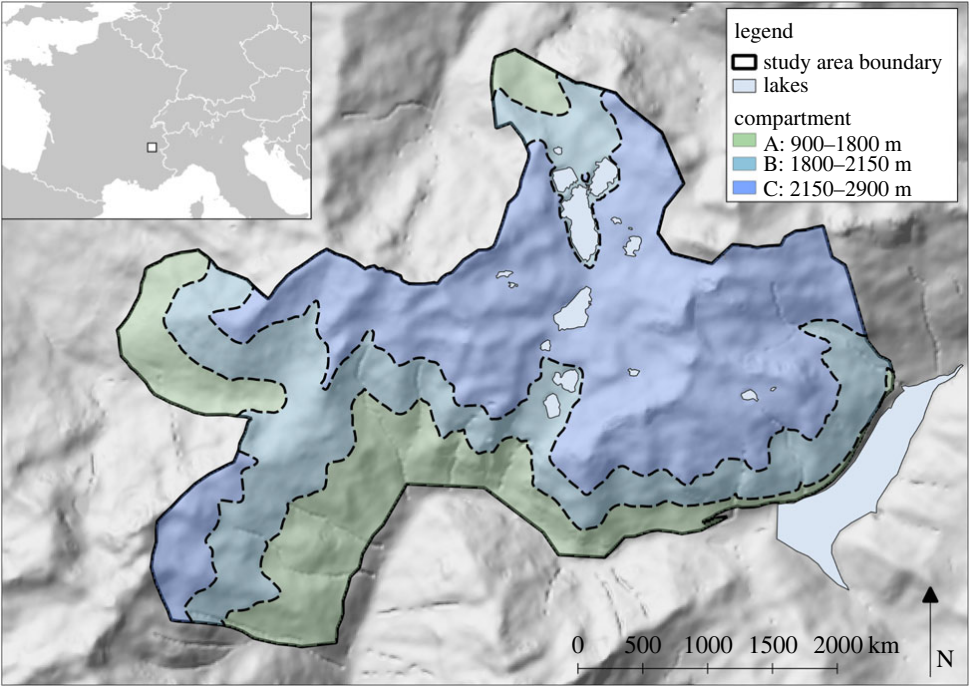
The study area showing the selected compartment elevation bands. Inset map shows the location of the study area in south-east France.

The study area, in the Belledonne Massif (6°4′ E, 45°13′ N, French Alps), covers 213 km^2^ with an elevation range between 900 and 2977 m. Since ibex were reintroduced to the study area in 1983, they have been continuously monitored through capture–mark–recapture methods by the Office Français de la Biodiversité [28]. The study area had a population of 800 free-ranging ibex (0.04 individuals per ha) and four herds of domestic sheep (*ca* 3600 individuals in total) grazed on mountain pastures between early July and early September (mean = 1.53 individuals per ha), using 2017–2019 estimates. Transhumance is practised and sheep are present in the study area only in the warmer summer months. Chamois (*Rupicapra rupicapra*) are found in sympatry with ibex, and two of the study sheep herds also included domestic goats (*Capra aegagrus hircus*) (*ca* 30 individuals). The goats were not included in the analysis because they are present in very small numbers. Chamois were not included because there are no data on their movements and space use in the study; however, they have overlapping parasite diversity with ibex and sheep [22]. The area was split into three compartments to represent different elevation bands: compartment A = 900–1800 m, compartment B = 1800–2150 m and compartment C = 2150–2900 m (figure 1). Elevation boundaries of compartments were delineated by ibex occupancy. This was determined using mean weekly elevation data for each individual animal via global positioning system (GPS) points with compartments selected when elevations were attributed to one third of host observations, see §2.3 for further details.

### 2.2. Climate data

Mean daily temperature and precipitation for the study area were extracted from the E-OBS gridded dataset at 0.1° resolution at an elevation of 1839 m [29]. Temperature and precipitation were adjusted for median host elevation in each compartment (A = 1661 m, B = 1915 m, C = 2300 m). Temperature was estimated to decrease by 0.0047°C m^−1^ [30] and precipitation was estimated to increase by 3.2 × 10^−4^ mm m^−1^ [31].

### 2.3. Host global positioning system location data

To measure host movement, GPS devices were deployed on ibex (*n* = 10 in 2017, *n* = 14 in 2018) and domestic sheep (*n* = 4 in 2017, *n* = 11 in 2018 and *n* = 18 in 2019). Each spring (May) captures of ibex were conducted using tele-anaesthesia or falling nets baited with salt to deploy devices, which were recovered the following April or May by radio-released drop-off. GPS devices were deployed on domestic sheep at the beginning of their grazing season (early July) and recovered when they returned from grazing pastures after *ca* eight weeks. Devices were programmed to record location every 2 h for ibex and every 30 min for domestic sheep. Elevation was then determined using individual location points overlayed on an elevation map. Elevations of GPS locations were averaged to give mean weekly elevation for individual ibex and sheep.

### 2.4. Host movement and elevation

Host density in each compartment was calculated using the estimated proportion of the total population of each host species observed in each compartment, calculated using the mean weekly elevation derived from the GPS locations. To predict the drivers of host elevation, mean weekly elevation was modelled for each host species separately. A generalized linear mixed effects model was used with the package ‘lme4’ [32], using R [33] with study year, mean weekly temperature and cumulative weekly precipitation as fixed effects, and individual (ID) as a random effect. The model also included season for ibex and week for sheep as a fixed effect. The global models were simplified using the *dredge* function in the R package ‘MuMIn’ [34] which uses corrected Akaike information criterion (AIC_c_) to rank model fits [35]. Models with ΔAIC_c_ ≤ 6 were retained for inference, excluding all models within which a nested alternative had a lower ΔAIC_c_ value. Model residuals were checked for a normal distribution. A binary logistic regression with temperature, year and week as explanatory variables was used to predict the presence or absence of sheep.

### 2.5. Model description

**Figure 2.**
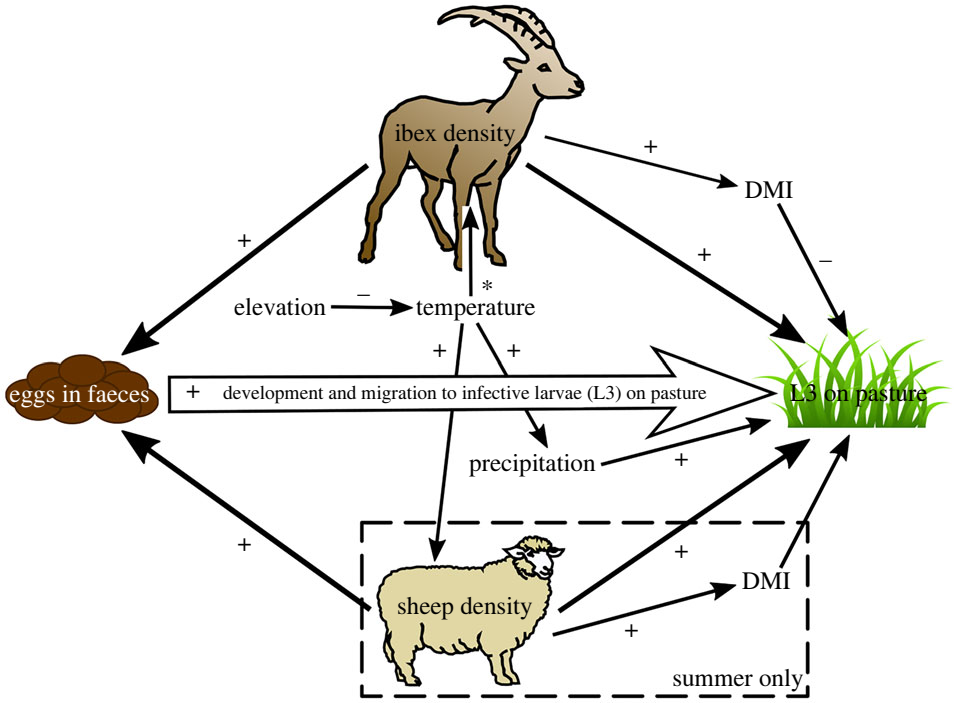
The hypothesized positive (+) and negative (−) interactions between the different elements of the study system which are incorporated into the model. Asterisk denotes the relationship between ibex density and temperature which varies by season. DMI, dry matter intake.

This study used the GLOWORM-FL model to predict the abundance of the free-living life cycle stages of *T. circumcincta* [36]*.* GLOWORM-FL estimates the temperature- and moisture-dependent development of parasitic GINs from eggs, after being deposited within host faeces, to L3, resulting in an estimate of the number of infective L3 present on grazing sites (see electronic supplementary material, S1; figure 2). The model was implemented in R v. 3.6.3 [33]. The model framework was adapted to account for differences in temperature, precipitation and host density between the defined elevational compartments. Scenario-based analyses were used to assess the effect of changing host species presence and density on predicted L3 abundance. This included (i) maintaining a constant ibex density throughout the year, (ii) varying ibex density throughout the year, and (iii) considering the additional impact of sheep grazing during the summer period.

Model output is the daily number of individuals per hectare for each parasite life stage; thus, the model predicts the density of L3 on the vegetation. Annual infection pressure in each compartment over the period occupied can be derived as the area under the curve (AUC), which was calculated using the trapezoid method with the *AUC* function in the ‘DescTools’ package [37]. A sensitivity analysis was conducted to determine the relationship between each of the main parameters and the model outcome (electronic supplementary material, S1).

### 2.6. Parameter estimation

Faecal GIN egg output was estimated from the available literature using host age, weight and faecal egg count (FEC) data. Ibex FEC reported in June to November (2013–2016) were used (mean = 249.1 eggs per gram (EPG), range = 100–741 EPG; [38]), and values outside this period (December to May) were uniformly randomly generated for each month assuming no temporal variation. The ibex population was estimated to have an average age of 7.86 years (range = 2–15 years; [13]). Due to the sexual dimorphism of ibex, weight was derived from two studies that report weight for males (75.7 ± 10.3 kg; [39]) and females (36.3 ± 10.9 kg; [40]). Average weight of ibex was 56.5 kg, calculated using a population sex ratio of 1.03 ± 0.17 [41].

For livestock, FEC reported for Merino D'Arles sheep measured in June, September and October (1982) while grazing on Alpine pastures were used (mean = 157 EPG, range = 16–250 EPG; [23]). Prior to grazing on pasture, sheep were treated with an anthelmintic, fenbendazole, for gastrointestinal and protostrongylid nematodes as well as rafoxanide for trematode parasites. Sheep FEC was input as measured by Gruner *et al*. [23] each month and interpolated for the intervening days. The herd of sheep was roughly 1 year old, and average weight was 30.25 ± 2.5 kg during the grazing period [23]. Faecal output was assumed to be the same for ibex and sheep (7.0 g of faecal dry matter/kg of body weight; [42]).

The GLOWORM-FL model has been validated using a range of laboratory and field observations of parasite development time and success [36]. Available data to validate the model output for this study system, however, were limited. To check the plausibility of the model output, we used field observations from Gruner *et al*. [23]. These pasture larval counts (L3 per kg dry herbage) were compared with the model output (electronic supplementary material, S1). To permit comparison with these L3 counts, the abundance of L3 in the model was converted to L3 per kg dry herbage (L3_h_ = L3/kgDM) by dividing L3_h_ by the biomass of dry herbage (970.52 kg ha^−1^, calculated from 242.63 g wet biomass m^−^^2^; [43]).

### 2.7. Climate change scenarios

Model simulations were performed using historic and projected future climate data to predict the effect of climate change on seasonal L3 availability at different elevations (see electronic supplementary material). For the historic climate observations, data were extracted from the E-OBS gridded dataset for a 30-year period (1976–2005) and the projected climate data were taken from the high emissions scenario (representative concentration pathway (RCP) 8.5) from the HADGEM-ES model output for a 30-year period (2066–2095) [44]. Mean daily temperature and total daily precipitation were adjusted for the elevation of each compartment as described above for historic data.

The simulations were run for four different scenarios: (i) historic: historic climate and observed host movement; (ii) projection 1: projected climate and observed host movement; (iii) projection 2: projected climate and predicted host movement (for ibex; observed sheep movement), and (iv) projection 3: projected climate and predicted host movement (both ibex and sheep). Predicted host movement was computed based on the drivers of host elevation and extrapolated for projected climate scenarios (§2.4).

## 3. Results

### 3.1. Host movement and elevation

**Figure 3.**
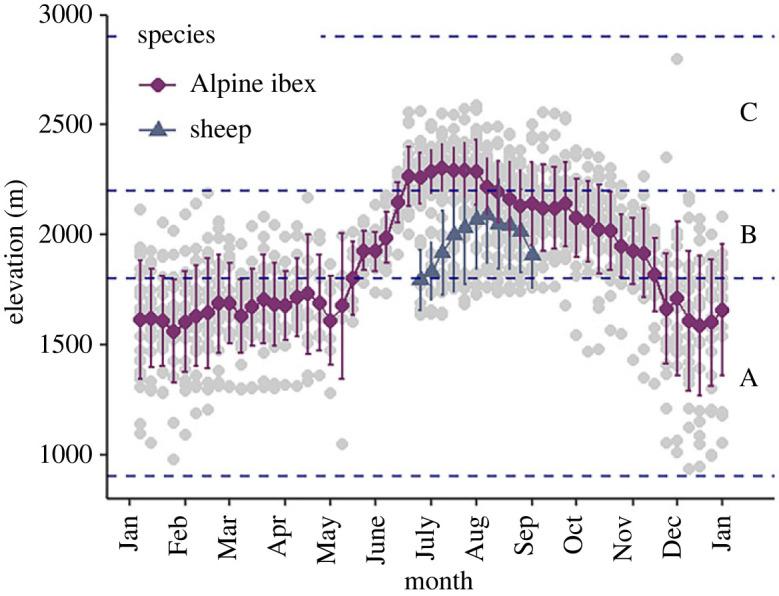
Mean weekly elevation of ibex and sheep in the study area with standard error bars. Grey points show each observation of individual weekly mean elevation. Blue dashed lines represent compartment boundaries.

Ibex elevation, derived from GPS locations, was recorded at a yearly mean elevation of 1923 m (s.d. ± 320 m), ranging from 935 to 2798 m. Weekly elevation of ibex varied significantly by season (likelihood ratio test: *χ*^2^ = 1033.8, d.f = 2, *p* < 0.001). Ibex were found at the highest elevation in summer (mean ± s.d. = 2208 ± 161 m) and lowest during winter (mean ± s.d. = 1625 ± 266 m; figure 3; table 1). Ibex were present in compartment B throughout the year and were only present in compartment A (low elevation) between September and June, and in compartment C (high elevation) between mid-June and the end of October. Sheep were recorded at a mean elevation of 1996 m (s.d. ± 223 m), ranging from 1633 to 2447 m. They were present in all three compartments during the eight-week grazing period (*ca* beginning of July to beginning of September), but most observations were recorded in compartment B (table 1).

**Table 1.** Host and environmental model parameters for each season and compartment. Sheep are only present for the first week in autumn.

season	compartment	mean weekly temperature (°C)	cumulative weekly rainfall (mm)	ibex density (ha^−1^)	ibex FEC (EPG)	sheep density (ha^−1^)	sheep FEC (EPG)
winter	A	3.35	3.75	0.037	195.12		
	B	0.53	3.84	0.012			
	C	−1.82	3.91	0.001			
spring	A	10.61	3.18	0.033	181.16		
	B	7.79	3.28	0.016			
	C	5.44	3.36	0.001			
summer	A	20.77	1.94	0.001	277.39	0.089	136.91
	B	17.95	2.00	0.016		0.144	
	C	15.60	2.05	0.034		0.084	
autumn	A	13.06	2.67	0.008	219.55	0.036	205.00
	B	10.24	2.74	0.029		0.072	
	C	7.89	2.79	0.013		0.009	

### 3.2. Effect of temperature on host movement

**Figure 4.**
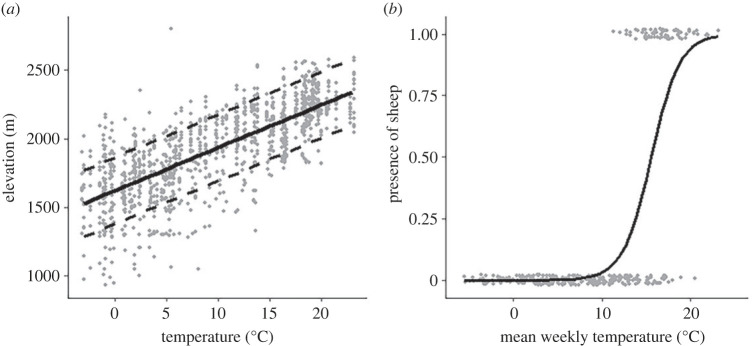
(*a*) Relationship between mean weekly temperature and observed elevation of ibex over the study period. The predicted relationship (solid line) and 95% prediction interval (dashed lines) are shown. (*b*) The presence or absence of sheep in the pastures as a function of temperature using a logistic regression.

The mean weekly elevation of ibex increased with higher temperatures and varied by season (table 2). Elevation was predicted to increase by 22.67 m per 1°C increase in temperature (figure 4*a*). Using projected climatic conditions (2066–2095), based on this elevation–temperature relationship, the mean weekly elevation of ibex was predicted to increase by 153 m (mean ± s.d. = 2076 ± 362). Sheep elevation did not change with temperature (table 2) but the presence of sheep, and therefore the timing of grazing, was significantly influenced by temperature (table 2; figure 4*b*). Sheep were present in the study area when the temperature was above 15.56°C.

**Table 2.** Top model set explaining (a) the elevation of ibex, (b) the elevation of sheep and (c) the presence of sheep, with all simpler models with ΔAIC_c_ < 6 included. The degrees of freedom (d.f.), amount of variation explained (*R*^2^), AIC_c_s and Akaike model weights are shown for each model.

model	temp.	precip.	year	season/week	d.f.	*R*^2^	ΔAIC_c_	weight
(a) ibex elevation	22.81		+	+	9	0.71	0.00	0.69
	22.26	−0.31	+	+	10	0.71	1.57	0.31
(b) sheep elevation			+	+	19	0.70	0.00	0.68
							
(c) presence of sheep	0.64	0.11			3	0.73	0.00	0.48
	0.61				2	0.71	0.08	0.46

### 3.3. Parasite transmission in ibex: effect of elevation

**Figure 5.**
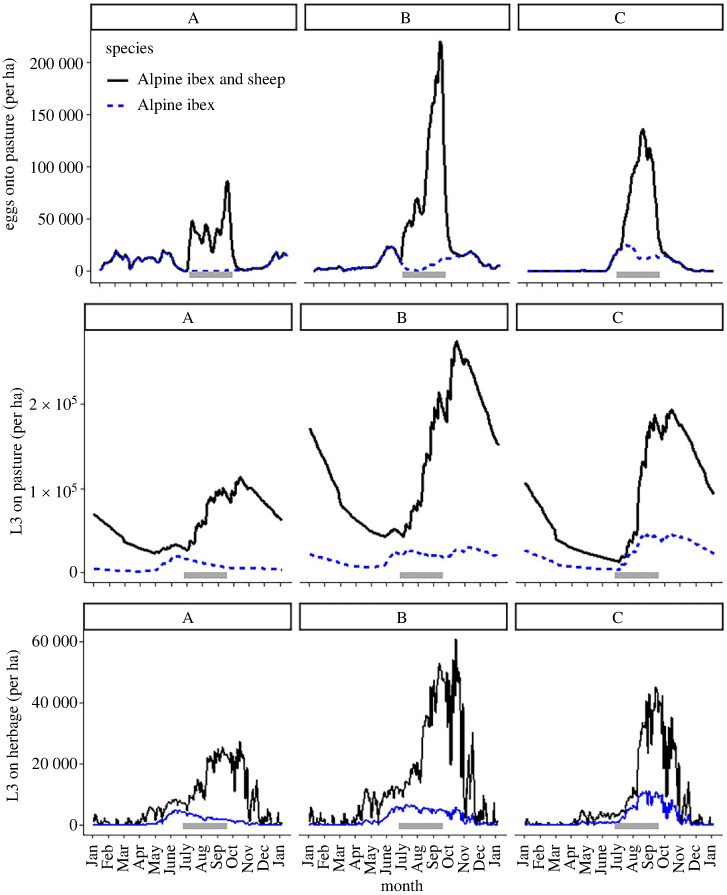
Output of the model showing egg output onto pasture, L3 on pasture (soil and herbage combined) and L3 on herbage per hectare, when only ibex are present (blue dotted line) and when ibex and sheep are present (black line) in each elevation compartment: (A: 900–1800 m, B: 1800–2150 m and C: 2150–2900 m). The grey bar shows the period that sheep are present.

Model simulations were performed with ibex density remaining constant through the year, and also when ibex density changed throughout the year due to varying grazing elevation. At constant host density, the annual infection pressure, as inferred from area under the L3_h_ abundance curve (AUC), decreased from 5.53 × 10^6^ L3 ha^−1^ year^−1^ (median L3_h_ [25th–75th percentile] = 1.75 [0.0027–2.74] × 10^4^ L3 ha^−1^) in the lowest compartment A to 5.32 × 10^6^ (1.51 [0–2.72] × 10^4^ L3 ha^−1^) and 4.79 × 10^6^ (1.11 [0–2.56] × 10^4^ L3 ha^−1^) in compartments B and C, respectively. When accounting for changes in host density at different elevations throughout the year, the annual infection pressure was predicted to be lowest in compartment A (AUC = 2.60 × 10^6^ L3 ha^−1^; median L3_h_ [25th–75th percentile] = 0.55 [0.36–1.00] × 10^4^ L3 ha^−1^) and higher in compartment B (6.79 × 10^6^ L3 ha^−1^; 2.05 [1.17–2.41] × 10^2^ L3 ha^−1^) and compartment C (8.15 × 10^6^ L3 ha^−1^; 2.16 [0.72–3.91] × 10^2^ L3 ha^−1^) (figure 5).

### 3.4. Parasite transmission in ibex and livestock

Inclusion of sheep in the model increased the predicted infection pressure significantly in each compartment, with the highest infection pressure in compartment B (table 3, figure 5). Of the total number of L3_h_ produced from both host species combined, sheep contributed 88%, 85% and 73% in each compartment A–C, respectively, despite being present in the system for only six to eight weeks of the year (table 3).

**Table 3.** Output of the parasite transmission models, showing the periods during which each elevation compartment was occupied by each host, the contribution towards total L3_h_ by each host in each compartment, measured by the total area under the L3_h_ density curve (AUC), and the L3_h_ exposure to the host, measured by the AUC for the period occupied by each host in each compartment. Compartments are in ascending order of elevation, A = lowest to C = highest.

host	compartment	period occupied by host (weeks)	contribution of L3_h_ (×10^6^ L3 ha^−1^ year^−1^)	L3_h_ exposure to host (×10^6^ L3 ha^−1^ year^−1^)
Alpine ibex	A	43	2.60	21.63
	B	51	6.79	45.15
	C	28	8.15	23.71
sheep	A	8	19.22	4.79
	B	8	40.12	7.81
	C	6	22.95	5.82

**Figure 6.**
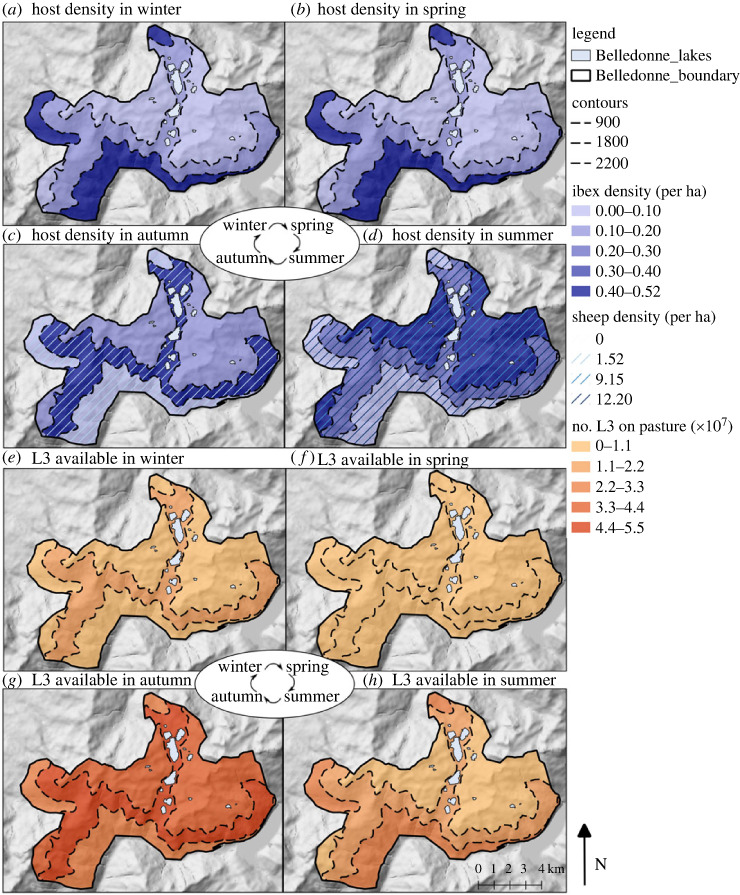
The change in host movement in the three compartments over the year (*a–d*), and the change in the number of L3 available on the pasture over the year (*e–h*).

The total exposure to L3_h_ of each host was highest for ibex, which were present throughout the year (Total AUC = 90.49 × 10^6^ L3 ha^−1^ year^−1^) and lower for sheep, which were present for a shorter period during the summer (Total AUC over grazing period = 18.42 × 10^6^ L3 ha^−1^ year^−1^; table 3). Ibex were predicted to be exposed to the highest infection pressure in the autumn when they returned from the highest elevations to compartment B, following grazing by sheep for an eight-week period (figure 5). Parasite life stages on pasture at this intermediate elevation continued to develop and survive after sheep had left, leading to a lag in L3 availability and peak levels when ibex arrived (figure 6).

To check the plausibility of the predicted L3_h_, the model output was compared with L3_h_ counted on pasture during sheep transhumance in a different area of the French Alps from Gruner *et al*. [23]. The L3_h_ per kgDM from the model output fitted within the standard deviation of pasture larvae counts for *T. circumcincta* L3 per kgDM, but was not significantly correlated (see electronic supplementary material, S1).

### 3.5. Effect of climate change

The projected climate data, at the location nearest to the study site and within the Alps, predicted more variable conditions with warmer temperatures and lower precipitation, especially in the summer (electronic supplementary material, figure S3). The mean projected elevation of ibex based on past temperature–elevation regression was 2076 ± 362 m, and the projected increase in elevation based on temperature preference was highest in the summer (206.5 m) and lowest in the spring (92.8 m). The projected period of sheep grazing was determined as the period when temperatures were consistently higher than 15.6°C. On this basis, sheep were predicted to be present grazing on the pastures for 24 weeks from the beginning of May to mid-October.

**Figure 7.**
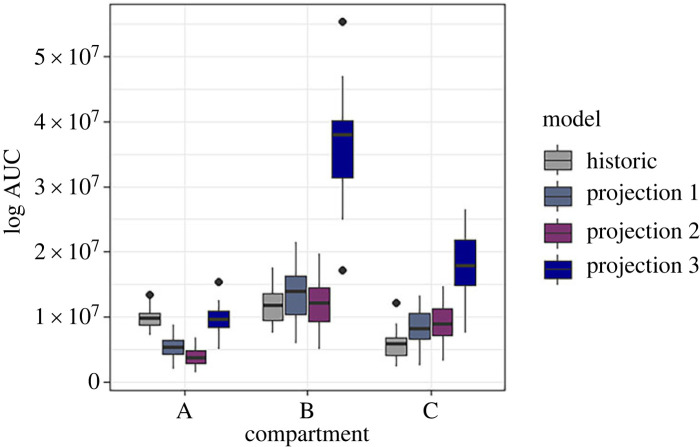
The estimated annual area AUC for the predicted density of L3_p_ (L3 ha^−1^ year^−1^) for the four simulated scenarios: historic, using the historic climatic and observed host movement data; projection 1, using the projected climate data based on the RCP 8.5 high emissions scenario and observed host movement data; projection 2, using the projected climate data and predicted ibex movement; projection 3, using the projected climate data and predicted ibex movement and livestock grazing patterns.

There was a significant predicted increase in the annual infection pressure of *T. circumcincta* for the projections under climate change, which varied by elevation compartment (*F*_11,342_ = 135.9, *p* < 0.001; figure 7). Infection pressure was highest in the model that predicted climate change with altered ibex movement and altered timing of livestock grazing (table 4). When altered ibex movement alone was considered, higher host elevation in summer attenuated the predicted increases in infection pressure, in compartments A and B. The largest increases in infection pressure were predicted in compartment B, except for the simulation which used projected climatic change and currently observed host movement. Climate projection simulations resulted in a higher number of L3 on the pasture at the end of the year than in historic climate scenarios, suggesting that a higher number of larvae would be able to overwinter and infect hosts at the beginning of the season the following year.

**Table 4.** The change in AUC for L3 density on pasture (L3_h_ ha^−1^ year^−1^), an index of total annual infection pressure, for each of the projected scenarios from the historic scenario for each compartment of the study, and the median and interquartile range in each compartment for each scenario.

model	compartment	ΔAUC (×10^7^ L3_h_ ha^−1^ year^−1^)	median	first quantile	third quantile
historic	A	—	9.86	8.83	10.65
	B	—	11.75	9.56	13.67
	C	—	5.85	4.18	6.77
	total	—	9.33	6.89	11.28
projection 1: climate	A	−4.52	5.38	4.31	6.42
	B	0.94	14.08	10.50	16.31
	C	2.37	8.18	6.54	10.58
	total	−1.20	8.13	5.55	11.97
projection 2: climate and ibex movement	A	−6.02	3.79	2.81	4.88
	B	−0.30	12.25	9.26	14.51
	C	2.91	9.01	7.09	11.39
	total	−1.33	7.99	4.77	11.39
projection 3: climate, ibex movement and livestock grazing period	A	−0.34	9.67	8.49	10.88
	B	21.88	38.11	31.44	40.36
	C	10.78	17.91	14.96	21.91
	total	8.29	17.61	10.71	30.37

## 4. Discussion

Understanding the impact of climate change on parasite transmission requires understanding the effect of interactions between host movement and climatic conditions on parasite infection potential. In this paper, we apply a model to understand transmission of the GIN species, *T. circumcincta*, in a mountain system where Alpine ibex overlap grazing areas with domestic sheep, and data on host movement and parasites are lacking. This model can be used in systems with environmental variation accounting for movement of multiple host species, to inform the management and conservation needs of wild species and livestock.

We found that host movement had a larger impact on parasite transmission than the direct impact of climate warming on parasite development rates. While climate warming would increase parasite development rates, we predicted this would be attenuated by ibex movement to higher elevations. As suggested by Brivio *et al*. [13], ibex will move to higher elevations with projected climate change, particularly during the warmer seasons. By forcing ibex to move to higher elevations in warmer projected scenarios, increases in transmission potential driven by climate change were attenuated because host density is lower in areas which are warmer and therefore more favourable for parasite development. On the other hand, ibex range is expected to be reduced as ibex are forced to higher elevations with less suitable habitat, leading to overcrowding [13]. This increased density of ibex could, in turn, increase parasite transmission in parts of its range.

By contrast, if livestock are grazed for longer periods due to warmer conditions, parasite transmission to ibex will increase regardless. Changes to the length of the sheep grazing season outweighed the attenuating effect of ibex movement on larval abundance and increased overall GIN infection pressure. Sheep were predicted to spend up to three times longer on pastures under projected climate change, and these results show that management of livestock will be important when considering the future of ibex.

Sheep, being more numerous, supplied the largest contribution to total parasite infection pressure, despite lower observed average infection levels and shorter grazing periods. Further, ibex were exposed to the highest infection pressure on return to intermediate elevations in autumn. Increasing elevation can reduce pressure from parasites due to cooler temperatures which slow development to infective parasite life stages [23,45]. However, vertical migration of ibex may in turn augment parasite transmission by visiting pastures when climate is suitable for parasite transmission. Sheep contributed to over 85% of the predicted L3 on herbage (L3_h_) at intermediate elevations despite only being present in the system for eight weeks, and the resulting peak of L3_h_ coincided with downward ibex migration from the highest elevations. Ibex trade off the best foraging opportunities to avoid high temperatures during the summer [46]; in autumn, they are likely to compensate by foraging at lower elevations as temperatures lower, increasing their exposure to parasites [47]. Since autumn is a critical time for ibex to gain body mass to enable over-winter survival [46,48–50], limiting infection pressure at this time could benefit individual fitness and population viability.

A similar model also suggested that grazing livestock generated seasonal peaks in contamination of pastures in autumn, leading to infection of horizontally migrating saiga antelopes, *Saiga tatarica*; and observed increases in parasite burdens over winter were consistent with predictions [21]. By contrast, parasite transmission modelling of pastures co-grazed by livestock and non-migratory bharal, *Pseudois nayaur*, in trans-Himalaya predicted shared transmission throughout the summer with no distinct peaks [18,19]. The complexity added in this study by strong seasonal host movements allows for highly focused interventions with disproportionate benefits for conservation which can account for likely future changes in system dynamics flexibly.

Since egg supply from sheep during summer grazing drives parasite risk to ibex health this should be reduced, for example by managing grazing timing and duration, reducing the number of sheep or reducing average egg output per sheep, for example by anthelmintic treatment or other antiparasitic interventions [51,52]. The model can identify periods and locations in which these egg inputs pose the greatest risk of ibex infection and could be applied to estimate the extent of the reduction needed to avoid increasing infection pressure on ibex in the future. Interventions should focus on these critical points, while aiming to be sustainable, due to the potential persistence and toxicity of some persistent anthelmintic drugs to a wide range of fauna, e.g. invertebrates, in an ecologically sensitive environment.

The model makes some assumptions that require qualification. It is assumed that responses of ibex and sheep grazing to climate change were independent of each other, but this might not be the case: Mason *et al*. [53], for example, found that avoidance of sheep by chamois was a stronger determinant of distribution under climate change than the direct effects of chamois temperature preferences. Moreover, current decision making for livestock grazing periods needs to be investigated further, to determine the drivers of decisions about the timing of grazing [54], whether climate change may alter the elevation at which sheep are grazing [45], and the routes by which altered grazing management might be implemented. We assume that historic associations between temperature and sheep presence will hold in future, although given that grass growth is limited by moisture and temperature, it seems plausible that climate change will increase forage availability and incentives to graze Alpine pastures over a longer season. Additionally, the availability of data for validation in this system is limited. The model can help prioritize data collection for model validation, specifically conducting pasture larvae counts and regular FECs in livestock. These data can then be incorporated to enable proactive and responsive, evidence-based management strategies at a local level. Government policies have already been suggested to be one of the most important factors for the future of ibex [13] and should arguably extend beyond grazing regulation to also include how livestock are managed to control parasites.

## 5. Conclusion

The dynamics of parasite transmission in multi-host mountain environments are difficult to disentangle, given the complex interactions between elevation, host movement and climate, compounded by climate change effects. Given data scarcity but solid understanding of factors driving parasite infective stage availability, parasite transmission modelling can address management concerns, for example targeting anthelmintic treatment timing in livestock, to manage parasites in wildlife. The modelling framework presented here can be extended to a multitude of different systems globally to investigate management options under conditions of host movement and environmental change. Notwithstanding additional complexities whose investigation might further refine understanding of parasite dynamics, insights from this study provide clear opportunities for application in support of wildlife and livestock health and population viability.

## Data Availability

Data and relevant code for this research work are stored in GitHub: https://github.com/ERDickinson0/ibex_gloworm_model and have been archived within the Zenodo repository: http://dx.doi.org/10.5281/zenodo.10257713 [55]. Supplementary material is available online [56].
